# Assessment of a rtPCR for the detection of virulent and benign *Dichelobacter nodosus*, the causative agent of ovine footrot, in Australia

**DOI:** 10.1186/s12917-018-1575-0

**Published:** 2018-08-29

**Authors:** Nickala Best, Lucas Zanandrez, Jacek Gwozdz, Eckard Klien, Nicky Buller, Robert Suter, Grant Rawlin, Travis Beddoe

**Affiliations:** 1 Department of Animal, Plant and Soil Science, Centre for AgriBioscience (AgriBio), La Trobe University, Bundoora, Melbourne, VIC Australia; 2 Federal University of Minas Gerais, Belo Horizonte, Minas Gerais Brazil; 3 Department of Economic Development, Jobs, Transport and Resources Centre for AgriBioscience (AgriBio), Victorian Government, Bundoora, Melbourne, VIC Australia; 4 DAFWA Diagnostics and Laboratory Services, Biosecurity and Regulation, Department of Agriculture and Food, South Perth, Western Australia; 5 Agriculture Services and Biosecurity Operations, Department of Economic Development, Jobs, Transport and Resources, Attwood, Victoria, Australia

**Keywords:** Australia, Benign, Footrot, Real-time polymerase chain reaction, Sensitivity, Sheep, Specificity, Victoria, Virulent

## Abstract

**Background:**

Ovine footrot is a highly contagious bacterial disease of sheep, costing the Australian sheep industry millions of dollars annually. *Dichelobacter nodosus*, the causative agent of footrot, is a gram-negative anaerobe classed into virulent and benign strains as determined by thermostability of their respective protesases. Current methods for detection of *D. nodosus* are difficult and time-consuming, however new molecular techniques capable of rapidly detecting and typing *D. nodosus* have been reported.

**Results:**

A competitive real-time PCR (rtPCR) method, based on the ability to detect a 2 nucleotide difference in the *aprV2* (virulent) and *aprB2* (benign) extracellular protease gene has been tested on Australian samples for determining detection rates, along with clinically relevant cut-off values and performance in comparison to the traditional culturing methods. The rtPCR assay was found to have a specificity of 98.3% for virulent and 98.7% for benign detection from samples collected. Sheep with clinical signs of footrot showed a detection rate for virulent strains of 81.1% and for benign strains of 18.9%. A cut-off value of a Ct of 35 was found to be the most appropriate for use in Victoria for detection of sheep carrying virulent *D. nodosus*.

**Conclusions:**

In summary, the rtPCR assay is significantly more capable of detecting *D. nodosus* than culturing, while there is no significant difference seen in virotyping between the two methods.

**Electronic supplementary material:**

The online version of this article (10.1186/s12917-018-1575-0) contains supplementary material, which is available to authorized users.

## Background

Ovine footrot is a highly contagious bacterial disease of sheep, causing lesions in the hoof and lameness [1]. The primary aetiologic agent of ovine footrot is *Dichelobacter nodosus*, a gram-negative anaerobe [2]. Many strains of *D. nodosus* exist consisting of multiple serogroups that are classified in Australia into virulent or benign based on extracellular protease activity. Infections with benign strains may appear as inflammation of the interdigital skin (interdigital dermatitis), while infections with virulent strains may vary from interdigital dermatitis to severe lesions with extensive necrosis and separation of the horn from the soft tissue [3, 4]. Footrot lesions are graded using a simple scoring system ranging from 0 (clinically healthy) to 5 (severe underrunning of the hard horn of the hoof) [3, 4]. The severity of lesions produced by virulent strains is reliant on environmental conditions, with temperatures of above 10 °C and consistent rainfall required for the full expression of virulence factors [5]. Subsequently, when environmental conditions are not optimal, infection with virulent strains may not be apparent clinically or it may present itself as mild infection imitating benign footrot [6]. The virulence potential of the *D. nodosus* strains may be determined by measuring the thermostability of serine proteases of isolates using the gelatin gel (gelatinase) test [7]. However, culture-based assays have been reported to have modest diagnostic power [8]. Furthermore, culture-based tests are labour-intensive and requires several weeks for the results to become available [9]. It has been reported that the acidic protease 2 (AprV2) plays a key role in virulence of *D. nodosus* [10]*.* Virulent strains have the *aprV2* gene encoding a thermostable protease. Benign strains have the gene *aprB2* encoding a thermolabile protease. The *aprV2* and *aprB2* alleles vary by a two-base pair substitution. This difference has been exploited in the probe design of a real-time polymerase chain reaction (rtPCR) assay [11]. Using this rtPCR assay, the presence of *D. nodosus* and its virulence can be determined within 1 day. The test is also capable of detecting both benign and virulent in the same clinical sample. There are limited data on the diagnostic performance of the rtPCR in sheep in Europe [12]. The objective of this paper was to evaluate the rtPCR assay with clinical samples collected from sheep in Victoria, Australia, with confirmed or suspected virulent footrot and sheep considered to be free of infection with *D. nodosus*.

## Methods

### Sample collection

The clinical samples were obtained retrospective from Victoria Government Veterinary Diagnostic unit collection. The samples were submitted by Victorian District Veterinary Officers, Animal Health Officers and private veterinary practitioners from the interdigital skin of lame sheep for routine diagnostic testing. Flocks were selected for this study if individual foot scores corresponding to sample labelling from individual sheep were provided, as well as the clinical history of the flock as determined by the submitting persons. Three hundred eighteen sheep from 10 flocks (#1 to 10) considered free of footrot, 170 sheep from 13 flocks (#12 to 24) with confirmed and/or suspected virulent footrot and 27 sheep from a closed flock with the history of virulent footrot that was deemed successfully eradicated. Prior to sampling, each sheep was examined and foot lesions scored and recorded. The interdigital skin of the foot with the highest score was sampled using two sterile swabs. One swab was placed into Stuarts Transport Media for culture and the second swab was placed into 800 μL of phosphate buffered saline (PBS) (8.1 mM Na_2_HPO_4_, 137 mM NaCl, 1.4 mM KH_2_HPO_4_ and 2.6 mM KCl) with 20 mM ethylenediaminetetraacetic acid (EDTA), pH 8.0), for rtPCR. The origin, breed, sex, age, foot score of the sheep sampled and their flock history are presented in Tables 1 and 2. Samples were kept at 4 °C after collection and sent the following day to AgriBio, 5 Ring Rd., La Trobe, Bundoora, Victoria, 3083. Samples were submitted for disease investigations pertaining to footrot.

**Table 1:** Descriptive characteristics of 11 Australian sheep flocks considered free of virulent footrot were sampled between June ‘15 and August ‘15 for evaluation of the specificity of an rtPCR for detection of virulent (*aprV2*) and benign (*aprB2*) protease genes of *D. nodosus*

Flock ID	No. animals sampled	Sampling date	Flock origin (shire or city)	Breed	Age	Sex	Comments/Flock history
1	18	02.06.2015	City of Broken Hill, NSW	Merino	Lambs	Mixed	Abbatoir line.
2	18	02.06.2015	Blayney Shire, NSW	Mixed	Mixed	Mixed	Abbatoir line.
3	19	02.06.2015	Shire ofArarat, Vic	Mixed	Rams	Male	Abbatoir line.
4	18	02.06.2015	Shire of Ararat, Vic	Merino	Mixed	Female	Abbatoir line.
5	19	02.06.2015	Shire of Ararat, Vic	Merino	Lambs	Mixed	Abbatoir line.
6	18	02.06.2015	City of Wagga Wagga, NSW	Merino	Ewes	Female	Abbatoir line.
7	18	02.06.2015	Southern Grampians Shire, Vic	Crossbreed	Ewes	Female	Abbatoir line.
8	55	16.06.2015	Yarra Ranges Shire, Vic	Coopworth crosses	Ewes	Female	Farm has had previous intermittent lameness, footrot has not been confirmed as the cause.
9	81	01.07.2015	Strathbogie Shire, Vic	Merino	Wethers	Male	Farm has no history of footrot, but lameness occasionally observed. Sheep footbathed late 2014.
10	54	17.07.2015	Wellington Shire, Vic	Merino	Wethers	Male	Well managed merino stud, no history of footrot. Wether had strayed into adjoining properties and had been cought and shorn 2 days prior to sampling.
11^a^	27	05.08.2015	East Gippsland Shire, Vic	Merino	Ewes	Female	Virulent footrot first introduced in the 80’s, treated by footbathing. Second footrot introduced in 1995; eradicated by footbathing, antibiotic regime and culling. A closed flock currently. No footbathing for ≥ 10 years.

^a^ Flock 11 has been excluded from specificity and sensitivity calculations because of its history

**Table 2:** Descriptive characteristics of 11 Australian sheep flocks considered having virulent footrot were sampled between October ‘14 and July ‘15 for evaluation of the specificity of an rtPCR for detection of virulent (*aprV2*) and benign (*aprB2*) protease genes of *D. nodosus*

Flock ID	No. animals sampled	Sampling date	Flock origin (shire or city)	Breed	Age	Sex	Comments/Flock history
12	26	27.07.2015	Wangaratta Rural City, Vic	Dorper	Rams	Male	Farm has a history of virulent footrot. Footbathing and foot pairing done frequently. Minimal lameness and lesions currently present. Sheep reviously footbathed in May 2015.
13	10	10.07.2015	East Gippsland Shire, Vic	Merino Cross	Mixed	Mixed	Footrot introduced December 2014 by purchased rams. Owners observed lameness of about 1 in 150 animals in March/April 2015.
14	36	23.06.2015	Mitchell Shire, Vic	Merino	Rams	Male	Farm suspected of virulent footrot, samples taken on a confirmatory visit.
15	10	13.10.2014	Strathbogie Shire, Vic	Merino	NR	Female	History of footrot and lameness in flock
16	10	13.10.2014	Greater Shepparton City, Vic	Merino cross	2.5 y	Female	A mob of South African Merino X Merino yearling ewes purchased in Nov-Dec 2013. Sheep had been on agistment. A few sheep lame when they arrived; footbathed 2–4 weeks before sampling.
17	10	14.10.2014	Shire of Newstead, Vic	Merino	Mixed	Female	Property has a footrot history - previously treated sucessfully with Footrite®. This season a recurrance of lameness, some mobs reached a 20%.
18	9	20.10.2014	Indigo Shire, Vic	Dorper	Adult	Female	Lameness in more than one foot. Lesions suggestive of benign footrot.
19	10	03.11.2014	Shire of Glenelg, Vic	NR	3.5 years	Female	Footrot appeared in June; source not determined, appears to be clinically aggressive; high prevalence of score 4/5 (20%+). These sheep last footbathed ~ 3 weeks before sampling.
20	10	04.11.2014	Southern Grampians Shire, Vic	NR	2 years	Female	Footrot has probably been present for a long time. Controlled by regular footbathing. Last month have had 30% of average long term rainfall for this time of the year. These sheep last footbathed ~ February 2014
21	10	17.11.2014	Bass Coast Shire, Vic	Droper	Mixed	Mixed	Many lambs, ewes and some rams reported lame with lesions very suggestive of footrot. Treated with footbath (formalin) and antibiotics. The lesions look in a process of healing but are still obvious (inflammation limited to interdigital space).
22	10	18.11.2014	Mitchell Shire, Vic	Merino	NR	NR	Virulent footrot for several years.
23	10	24.11.2014	Southern Grampians Shire, Vic	NR	Adult	Female	Footrot appeared in June (source not determined) with high prevalence of score 4/5 (20%+). Last 3 months have had 30% of average long term rainfall for this time of the year. These sheep last footbathed ~ 3 weeks earlier.
24	9	01.12.2014	Colac Ottway Shire, Vic	Crossbred	Adult	Female	10 of 15 seep examined had feet lesions scored ≥ 2

### Culturing of *D. nodosus*

Swabs collected from sheep from flocks 1 to 14 were plated at AgriBio one to 2 days after collection onto 4% (*w*/*v*) agar with 3% (w/v) ground hoof media (Footrot Reference Laboratory, Department of Agriculture, Perth, Australia) and anaerobically incubated at 37 °C for 7 days. Plates were examined for *D. nodosus* growth and suspect colonies subcultured and gram stained as described elsewhere [9]. *D. nodosus* isolate A198 (*aprV2*) (AC: 6466), virulent control, and isolate C305 (*aprB2*) (AC: 6465), benign control, were obtained from the Footrot Reference Laboratory and grown concurrently with sample plates. Isolates morphologically consistent with *D. nodosus* were sent on ice to the Footrot Reference Laboratory for gelatinase testing. Swabs collected from sheep from flocks 15 to 24 were sent on ice by overnight courier to the Footrot Reference Laboratory, Perth, Western Australia for culture and gelatinase testing.

### DNA extraction

DNA was extracted from swabs using a commercial extraction kit (MagMAX™ − 96 Viral RNA isolation kit, Ambion, Austin, USA) and purification system (Kingfisher-96 magnetic particle handling system, Thermo Fisher Scientific, Finland). Swabs from two positive culture controls were used as positive extraction controls. Swabs collected from sheep from flocks 1 to 14 were subject to DNA extraction on two separate occasions (runs).

### *aprV2/B2* rtPCR

Primers, probes and cycling conditions as described by Stauble et al.*,* 2014 were used [11]. A commercial rtPCR kit (AgPath-ID™ One Step RT-PCR Kit, Ambion, Austin, USA) was used as master mix according to manufacturer’s instructions, with final concentrations of 300 nM primers, 100 nM DnAprTM-vMGB, 250 nM DnAprTM-bMGB and 5 μL of template DNA. Primers and probes were synthesised commercially (Primers and probes, Applied Biosystems, California, USA) Reactions were carried out and analysed (7500 Fast Real-Time PCR System, Life Technologies) with a set threshold of 0.05. Two DNA extracts derived from sheep from flocks 1 to 14 were assayed by the rtPCR in two separate runs. Singular DNA extracts derived from sheep from flocks 15 to 24 were assayed by the rtPCR in one run.

Positive extraction controls from live cultures of isolate A198 and C305, and purified and isolated genomic DNA from the same culture isolates were used as rtPCR controls in each run. The rtPCR run was considered valid when results obtained in rtPCR controls were concordant. Results are reported as cycling threshold (Ct) values, the point at which the sample signal exceeds the threshold of 0.05. Samples producing a probe-specific fluorescent signal were defined as being positive. The effect of two cut-off values; Ct < 40 and Ct < 35, on the rtPCR detection rate and specificity, was assessed.

### Data analysis

Considering the culture/gelatinase method lacks adequate diagnostic accuracy and both the virulent and benign strains of *D. nodosus* may produce subclinical or mild, clinically unapparent infection, detection rates of the *aprB2* rtPCR and *aprV2* rtPCR were calculated using data obtained from 135 sheep with foot lesions and 35 healthy sheep from the 13 flocks with confirmed or suspected virulent footrot. Because of the lack of the gold diagnostic standard, the specificities of the *aprB2* rtPCR and *aprV2* rtPCR were calculated using data derived only from the 297 healthy sheep from the 10 flocks considered to be free of footrot. Twenty-one sheep with foot lesions scored 1 and 2 were excluded from the specificity calculations. Both the detection rate of clinically infected animals and specificity of the rtPCR were calculated for cut-offs of Ct of 35 and 40 values respectively, using data obtained in the first rtPCR run. Descriptive statistics were performed with Microsoft Excel 2010, Microsoft Corporation, Redmond, WA. Further analyses including Cohen’s kappa statistic (agreement between rtPCR runs, agreement between gelatinase gel test and rtPCR virulence designations), using the Altman scheme where ≤0 = worse than chance alone, < 0.20 = poor, 0.21–0.40 = fair, 0.41–0.60 = moderate, 0.61–0.80 = good, and 0.81–0.99 = very good, 1.00 = perfect and McNemar’s test for comparisons of foot lesions vs. rtPCR, culture vs. rPCR and rtPCR run vs. rtPCR run were also performed withGraphPad Prism version 4.01 for Windows, GraphPad Software, La Jolla, CA.

## Results

The rtPCR results in relation to foot scores and culture/gelatinase test results obtained in 10 flocks considered free of footrot, 13 flocks with confirmed and/or suspected virulent footrot and a closed flock (#11) with the history of eradicated virulent footrot are presented in Table 3.

**Table 3:** Clinical scores and results of *D. nodosus* culture, gelatinase test and rtPCR (aprB2/aprV2 positive, two cut offs, duplicate runs) in 318 sheep from flocks considered to be free of footrot (clinically healthy), 170 sheep from flocks considered having virulent footrot (clinically affected) and 27 sheep from a closed flock (#11) that apparently eradicated virulent footrot more than 10 years ago. Flocks were sampled from October 2014 to August 2015

	Flock ID	No. sheep tested	Results											aprB2 rtPCR^d^				aprV2 rtPCR^d^			
			Clinical Score^b^							Culture^c^	Gelatinase test^d^										
														Run 1		Run 2		Run 1		Run 2	
			0	1	2	3	4	5	Mean		Benign	Virulent	Undetermined	Cut-off		Cut-off		Cut-off		Cut-off	
														40	35	40	35	40	35	40	35
Clinically healthy	1	18	18	–	–	–	–	–	0	–	–	–	–	–	–	–	–	–	–	–	–
	2	18	18	–	–	–	–	–	0	1	–	–	1	–	–	–	–	2	2	2	2
	3	19	17	–	1	–	1	–	0.1	–	–	–	–	–	–	1	–	–	–	–	–
	4	18	11	1	6	–	–	–	0.6	–	–	–	–	–	–	4	–	–	–	–	–
	5	19	19	–	–	–	–	–	0	–	–	–	–	–	–	2	–	–	–	–	–
	6	18	11	3	1	1	–	2	0.8	–	–	–	–	–	–	–	–	5	4	6	4
	7	18	16	2	–	–	–	–	0.1	–	–	–	–	–	–	–	–	–	–	–	–
	8	55	55	–	–	–	–	–	0	–	–	–	–	5	1	1	1	3	2	4	2
	9	81	81	–	–	–	–	–	0	2	2	–	–	5	3	4	3	2	–	5	–
	10	54	51	3	–	–	–	–	0.1	–	–	–	–	8	–	–	–	2	1	2	1
Subtotal		318	297	9	8	1	1	2		3	2	0	1	18	4	#	4	14	9	19	9
	11^a^	27	11	16	–	–	–	–	0.6	–	–	–	–	16	10	20	13	–	–	–	–
Clinically affected	12	26	14	12	–	–	–	–	0.5	7	–	5	2	–	–	–	–	26	25	26	26
	13	10	1	3	–	5	1	–	2.2	4	2	2	–	5	3	3	3	9	6	9	6
	14	36	20	8	3	–	–	5	1.1	2	–	2	–	6	5	6	5	17	14	21	13
	15	10	–	–	1	–	2	7	4.5	2	–	2	–	–	–	ND	ND	10	9	ND	ND
	16	10	–	–	4	3	2	1	3	5	–	5	–	–	–	ND	ND	10	10	ND	ND
	17	10	–	–	4	2	4	–	1	10	4	6	–	4	4	ND	ND	6	6	ND	ND
	18	9	–	9	–	–	–	–	4.9	3	–	3	–	–	–	ND	ND	9	9	ND	ND
	19	10	–	–	–	–	1	9	2.7	3	–	3	–	–	–	ND	ND	9	9	ND	ND
	20	10	–	–	4	5	1	–	1.1	3	2	1	–	10	10	ND	ND	10	10	ND	ND
	21	10	–	9	1	–	–	–	3.5	–	–	–	–	2	2	ND	ND	2	1	ND	ND
	22	10	–	–	1	5	3	1	3.3	6	1	5	–	–	–	ND	ND	10	10	10	10
	23	10	–	–	–	–	10	–	4	–	–	–	–	–	–	ND	ND	10	10	ND	ND
	24	9	–	–	5	2	2	–	2.6	–	–	–	–	–	–	ND	ND	9	8	ND	ND
Subtotal		170	35	41	23	22	26	23		45	9	34	2	27	24	9	8	137	127	66	55

^a^ Flock 11 has been excluded from specificity and sensitivity calculations because of its history

^b^ Results expressed as a number of sheep in which the highest rated foot was in the particular score

^c^ Results expressed as a number of sheep from which an organism morphologically consistent with *D. nodosus* was isolated

^d^ Results expressed as a number of sheep that tested positive

For all data, Ct’s of 40 and 35 were investigated as suitable cut-off values to interpret positive results. From data collected through the clinically healthy trial, the use of a Ct of 40 showed poor discrimination between healthy and clinically affected sheep in both *aprV2* and *aprB2* results (*p* > 0.005, both, Table 4). When using a Ct of 35 and under to indicate positive rtPCR results, detection of footrot using rtPCR differed significantly from using clinical signs alone (*p* = 0.0014 for *aprB2*, and *p* = 0.019 for *aprV2*). The agreement between repeated runs at Ct of 40 for virulent results was good (kappa = 0.731), and perfect when using a Ct of 35 (kappa = 1). A similar result is seen for the benign rtPCR results, with a reasonable agreement for a Ct of 40 between repeats (kappa = 0.302), and a highly significant agreement when using a Ct of 35 (kappa = 1) (Table 4). In the data from clinically affected animals, there was a significant difference between numbers of positive results obtained by the *aprV2* rtPCR and *aprB2* rtPCR at a cut-off of 40 and 35 respectively, in the clinically affected (*n* = 135) and healthy (*n* = 35) sheep. Using a Ct of 35 resulted in a better agreement between replicates of the same sample (Table 5). Results using a Ct of 35 only will be reported further due to the increased agreement between repeats.

**Table 4:** Results of duplicate runs at cut offs Ct 40 and 35 for *aprV2* and *aprB2* rtPCR in 297 clinically healthy sheep and 21 sheep with foot lesions from 10 flocks considered free of footrot

Foot lesion	Cut off 40				Cut off 35			
	Run 1 + ve	Run 1 -ve	Run 2 + ve	Run 2 -ve	Run 1 + ve	Run 1 -ve	Run 2 + ve	Run 2 -ve
*aprB2*								
Positive	0	21	2	19	0	21	0	21
Negative	18	279	10	287	4	293	4	293
Specificity		93.90%		96.60%		98.70%		98.70%
McNemars two tailed *p* value		0.7488		0.1374		0.0014		0.0014
*aprB2 cut off agreement*								
Run 2 + ve	5	7			4	0		
Run 2 -ve	13	293			0	314		
Kappa	0.302	“fair”			1	“perfect”		
*aprV2*								
Positive	4	17	4	17	4	17	4	17
Negative	10	287	15	282	5	292	5	292
Specificity		96.60%		94.90%		98.30%		98.30%
McNemars two tailed *p* value		0.2482		0.8597		0.019		0.019
*aprV2 cut off agreement*								
Run 2 + ve	12	7			9	0		
Run 2 -ve	2	297			0	309		
Kappa	0.713	“good”			1	“perfect”		

Specificity is shown along with *p*-value for McNemar’s test for independence between lesion score and rtPCR result, and kappa statistic for agreement between rtPCR runs

**Table 5:** Results from two cut offs of Ct 40 and 35 from the *aprB2* rtPCR and *aprV2* rtPCR obtained from two runs, in 72 sheep randomly sampled from 3 flocks considered having virulent footrot

Foot lesion	Cut off 40				Cut off 35			
	Run 1 + ve	Run 1 -ve	Run 2 + ve	Run 2 -ve	Run 1 + ve	Run 1 -ve	Run 2 + ve	Run 2 -ve
*aprB2*								
Positive	9	28	8	29	7	30	7	30
Negative	2	33	1	34	1	34	1	34
Overall % positive		15.30%		12.50%		11.10%		11.10%
McNemars two tailed *p* value		< 0.0001		< 0.0001		< 0.0001		< 0.0001
*aprB2 cut off agreement*								
Run 2 + ve	8	1			8	0		
Run 2 -ve	3	60			0	64		
Kappa	0.768	“good”			1	“perfect”		
*aprV2*								
Positive	33	4	33	4	30	7	29	8
Negative	19	16	23	12	15	20	16	19
Overall % positive		72.20%		77.80%		62.50%		62.50%
McNemars two tailed *p* value		0.0035		0.0005		0.1356		0.153
*aprV2 cut off agreement*								
Run 2 + ve	51	5			44	1		
Run 2 -ve	1	15			1	26		
Kappa	0.779	“good”			0.941	“very good”		

The *p*-value for McNemar’s test for independence between lesion score and rtPCR result is shown along with kappa statistic for agreement between rtPCR runs

Using 297 animals from historically healthy farms scored 0, specificity was calculated to be 98.3% for *aprV2* detection, and 98.7% for *aprB2* detection when using a Ct of 35 as the cut-off. From these flocks, 318 animals were tested in total, with 21 scored 1–5 (Table 3). One sheep of the 318 from the flocks considered free of footrot yielded growth of *D. nodosus* of undetermined gelatinase profile. This sheep (Flock 2) also produced strong reactions (Ct ≤ 31) in the *aprV2* rtPCR in both runs. Two other sheep (Flock 9) tested positive for the benign strain of *D. nodosus* by culture/gelatinase test and also by the *aprB2* rtPCR in both runs (Ct ≤ 33.74). One of these two sheep gave also a weak reaction (Ct 38.39) in the *aprV2* rtPCR in one run. All three sheep that yielded growth of *D. nodosus* and tested positive by the *aprV2* rtPCR and/or *aprB2* rtPCR were clinically healthy (Table 3).

From the 170 animals clinically affected or suspected, 135 were scored 1 or above and considered clinically affected, while 35 were scored 0. The rtPCR produced positive virulent results in 112 of the 135 clinically affected sheep, giving a detection rate of 83% when using a Ct of 35, or 81.1% overall when using all 170 animals (Table 3).

In comparison, 45 animals from the 170 in the affected group had *D. nodosus* isolates successfully obtained and 43 had the gelatinase test performed (Table 6). There was a significant difference between the two methods capabilities to detect virulent *D. nodosus* (*p* < 0.0001). Comparing the gelatin gel designation of virulence to the rtPCR results, there is no significant difference between the two tests when using 37 of the isolates (McNemars Test, *p* = 0.479). Samples that tested positive for *aprV2* and *aprB2* via rtPCR and had *D. nodosus* successfully isolated were excluded from the above calculation as no sample had more than one isolate obtained. None of the 135 clinically affected sheep and 35 clinically healthy sheep tested negative by the *aprV2* rtPCR but positive for the virulent strain of *D. nodosus* by the culture/gelatinase test. The agreement between results produced by the *aprV2* rtPCR and that obtained by the culture/gelatinase test ranged from fair (Kappa = 0.2–0.222) to poor (kappa = 0.082–0.158) (Table 7). In total, when the affected flock samples were cultured, an overall detection rate of 25% was obtained (Table 7).

**Table 6:** Gelatinase gel test and rtPCR results for 45 individual samples within the clinically affected data set that successfully had *D. nodosus* isolated, where S is thermostable (virulent) and U is thermolabile (benign)

Flock ID	Score^a^	Gelatinase gel test	rtPCR
12	1	S	*aprV2*
	1	S	*aprV2*
	0	S	*aprV2*
	0	NA	*aprV2*
	0	S	*aprV2*
	0	NA	*aprV2*
	0	S	*aprV2*
13	1	U	*aprV2* and *aprB2*
	1	S	*aprV2* and *aprB2*
	3B	U	*aprV2*
	3C	S	*aprV2*
14	5	S	*aprV2*
	1	S	*aprV2* and *aprB2*
15	5	S	*aprV2*
	5	S	*aprV2*
16	3	S	*aprV2*
	2	S	*aprV2*
	2	S	*aprV2*
	3	S	*aprV2*
	4	S	*aprV2*
17	4	S	*aprV2*
	2	S	*aprV2*
	2	U	*aprB2*
	4	U	*aprB2*
	4	U	*aprB2*
	3	U	*aprB2*
	3	S	*aprV2*
	2	S	*aprV2*
	4	S	*aprV2*
	2	S	*aprV2*
18	1	S	*aprV2*
	1	S	*aprV2*
	1	S	*aprV2*
19	5	S	*aprV2*
	5	S	*aprV2*
	5	S	*aprV2*
20	3A	S	*aprV2* and *aprB2*
	2	U	*aprV2* and *aprB2*
	3B	S	*aprV2* and *aprB2*
22	3A	S	*aprV2*
	3A	U	*aprV2*
	4	S	*aprV2*
	3A	S	*aprV2*
	3	S	*aprV2*
	4	S	*aprV2*

^a^Foot scores are according to Stewart et al., 1983 [4]. An additional file describes the foot scoring in more detail (see Additional file 1)

**Table 7:** Comparisons of identification of *D. nodosus* by the *aprV2* rtPCR and culture/gelatinase gel test from subsets of presence/absence or both of foot lesions from the clinically affected flocks, consisting of 135 clinically affected sheep and 35 clinically healthy sheep

Flocks	Sheep clinical status (foot lesions)	No. sheep tested	aprV2 PCR Run/Cut-off	Culture Gelatinase Thermostable (Virulent) vs aprV2 PCR						Percentage positive results	
				Concordant results		Culture +ve	PCR + ve	*p* value	Kappa	Culture	aprV2 PCR
				+ve	-ve	PCR -ve	Culture -ve				
12 to 24	+ve	135	Run 1 / 40	31	17	0	87	< 0.0001	0.082	23.0%	87.4%
12 to 24	+ve	135	Run 1 / 35	31	20	0	84	< 0.0001	0.1	23.0%	85.2%
12 to 14	-ve	35	Run 1 / 40	3	16	0	16	0.0002	0.146	8.6%	54.3%
12 to 14	-ve	35	Run 2 / 40	3	12	0	20	< 0.0001	0.093	8.6%	65.7%
12 to 14	-ve	35	Run 1 / 35	3	20	0	12	0.0015	0.222	8.6%	42.9%
12 to 14	-ve	35	Run 2 / 35	3	19	0	13	0.0009	0.2	8.6%	45.7%
12 to 14	-ve & + ve	72	Run 1 / 40	9	20	0	43	< 0.0001	0.104	12.50%	72.20%
12 to 14	-ve & + ve	72	Run 2 / 40	9	16	0	47	< 0.0001	0.078	12.50%	77.80%
12 to 14	-ve & + ve	72	Run 1 / 35	9	27	0	36	< 0.0001	0.158	12.50%	62.50%
12 to 14	-ve & + ve	72	Run 2 / 35	9	27	0	36	< 0.0001	0.158	12.50%	62.50%
12 to 24	-ve & + ve	170	Run 1 / 40	34	33	0	103	< 0.0001	0.114	20.0%	80.6%
12 to 24	-ve & + ve	170	Run 1 / 35	34	40	0	96	< 0.0001	0.143	20.0%	76.5%

The *p*-value for McNemar’s test for independence between culture gelatinase thermostable (Virulent) and *aprV2* PCR result is shown. Detection rates of both culture and rtPCR are presented as percentage positive results

There was a significant difference (*p* ≤ 0.0015) between numbers of animals tested positive for the virulent strain of *D. nodosus* by the *aprV2* rtPCR and culture/gelatinase test among the 35 clinically healthy sheep. In this group, the *aprV2* rtPCR gave positive results in 15 (42.9%) and 16 (45.7%) of clinically healthy animals at a Ct of 35 cut-off value in run 1 and run 2, respectively, whereas the gelatinase test gave positive results in 3 of the healthy animals (Table 7).

The same method was applied to *aprB2* rtPCR positive samples, resulting in a detection rate of 18.9%, with 23 of 135 clinically affected animals (17%) positive for *aprB2* via rtPCR. Using culturing and the gelatin gel test, 9 (6.7%) of the 135 clinically affected animals tested positive for benign *D. nodosus*. Again, there was a significant difference between the two methods capabilities to detect benign *D. nodosus* (*p* = 0.0022). Two of the 135 clinically affected sheep tested positive for the benign strain of *D. nodosus* by the culture/gelatinase test but negative by the *aprB2* rtPCR. The agreement between results produced by the *aprB2* rtPCR and that obtained by the culture/gelatinase test ranged from fair (Kappa = 0.336–0.378) to poor (kappa = 0–0.163) (Table 8).

**Table 8:** Comparisons of identification of *D. nodosus* by the *aprB2* rtPCR and culture/gelatinase gel test from subsets of presence/absence or both of foot lesions from the clinically affected flocks, consisting of 135 clinically affected sheep and 35 clinically healthy sheep

Flocks	Sheep clinical status (foot lesions)	No. sheep tested	aprB2 PCR Run and/or Cut-off	Culture Gelatinase Thermolabile (Benign) vs aprB2 PCR						Percentage positive results	
				Concordant results		Culture +ve	PCR + ve	*p* value	Kappa	Culture	aprB2 PCR
				+ve	-ve	PCR -ve	Culture -ve				
12 to 24	+ve	135	Run 1 / 40	7	108	2	18	0.0008	0.348	6.7%	18.5%
12 to 24	+ve	135	Run 1 / 35	7	110	2	16	0.0022	0.378	6.7%	17.0%
12 to 14	-ve	35	Run 1 / 40	0	33	0	2	0.4795	0	0.0%	5.7%
12 to 14	-ve	35	Run 2 / 40	0	34	0	1	1	0	0.0%	2.9%
12 to 14	-ve	35	Run 1 / 35	0	34	0	1	1	0	0.0%	2.9%
12 to 14	-ve	35	Run 2 / 35	0	34	0	1	1	0	0.0%	2.9%
12 to 14	-ve & + ve	72	Run 1 / 40	1	60	1	10	0.0159	0.112	2.8%	15.3%
12 to 14	-ve & + ve	72	Run 2 / 40	1	62	1	8	0.0455	0.143	2.8%	12.5%
12 to 14	-ve & + ve	72	Run 1 / 35	1	63	1	7	0.0771	0.163	2.8%	11.1%
12 to 14	-ve & + ve	72	Run 2 / 35	1	63	1	7	0.0771	0.163	2.8%	11.1%
12 to 24	-ve & + ve	170	Run 1 / 40	7	141	2	20	0.0003	0.336	5.3%	15.9%
12 to 24	-ve & + ve	170	Run 1 / 35	7	141	2	17	0.0013	0.376	5.3%	14.1%

The *p*-value for McNemar’s test for independence between culture gelatinase thermostable (benign) and *aprB2* PCR result is shown. Detection rates of both culture and rtPCR are presented as percentage positive results

Among the 35 clinically healthy sheep, there was no significant difference (*p* = 1) between numbers of animal’s positive for the benign strain of *D. nodosus* by the *aprB2* rtPCR and culture/gelatinase test. In this group, the *aprB2* rtPCR at a Ct of 35 gave a positive reaction in 1 (2.9%) animal. None of the 35 clinically healthy sheep tested positive for the benign strain of *D. nodosus* by the culture/gelatinase test.

## Discussion

New molecular techniques capable of rapidly detecting and typing *D. nodosus* are required for improved diagnostics in Australia. A rtPCR method, capable of detecting and discrimination virulent *D. nodosus* strains, has been developed under European conditions. This rtPCR was assessed on Australian samples for detection rates, along with clinically relevant cut-off values. Ct values of 40 and 35 for positive identification cut off were investigated with regards to repeatability between runs, with signals above Ct 35 showing more discrepancies than those below 35. This is common in rtPCR assays, often with results past the Ct of 35 commonly seen as outliers [13]. In addition to higher specificities and more significant results at a Ct of 35 for positive samples (*aprV2*, *p* = 0.019, *aprB2, p* = 0.002, McNemar’s two-tailed *p*-value), no animal scored 1 or above returned a positive result above a Ct of 35, supporting the use of a cut-off set at 35 in diagnosing animals with some form of the disease. Interestingly, 6 animals that are clinically negative were *aprV2* positive after lowering the cut-off. This could suggest that these animals be monitored for clinical signs of footrot when favourable environmental conditions occur. They may potentially be asymptomatic carriers depending on circumstance, with the ability to re-infect the flock [14, 15].

The diagnostic power of rtPCR was assessed using the current clinical scoring system to judge if an animal was diseased, or free from disease, as this is the currently accepted method of diagnosis in Victoria. The detection of *D. nodosus* on 3 animals within the healthy population, by both rtPCR and culturing, suggests the presence of infection but not the disease [15]. As the population used for the detection of *D. nodosus* on clinically affected animals was deliberately chosen for clinical virulence, a low detection rate for benign *D. nodosus* was anticipated. From the animals that had the *aprB2* gene detected in the rtPCR, 81% were in association with a co-infection, where *aprV2* was also detected. As the virulent form of footrot is the clinical disease of interest, no additional investigation into a purely benign population was conducted. The reported overall detection rate for *aprV2* at a Ct of 35 is conservative, with an increase being seen when using a Ct of 40 (89.7%), and also when only using animals scored 3–5, which are traditionally considered virulent [9]. However, as the population used had confirmed or suspected of virulent footrot, the full range of scores were used to indicate the presence of disease. As *D. nodosus* is found in the sheep hoof environment and its presence does not always result in disease [16], it was expected that a number of the clinically negative sheep, throughout the whole data set, would return a positive result due to the nature of the sheep hoof and its environment. Further research monitoring the development of disease in association with environment on these animals may provide insights into the usefulness of *D. nodosus* detection prior to lesion formation, and therefore have practical management applications.

Time taken to receive a result using the rtPCR was typically within 1 day of sample collection or receipt. This is in comparison to the average of 2 to 4 weeks taken for a result when using the culturing method, while the rtPCR also provided better detection of *D. nodosus* from the samples collected. The advantage this provides would allow for a timelier confirmation of the presence of *D. nodosus*, confirming the clinical symptom is in association with the presence of bacteria. The gelatinase test relies on the phenotypic expression of proteases and the associated thermostability of those produced, and the culturing of *D. nodosus* is difficult and requires specialist skills and media. There are also inherent disadvantages to using this method, including the chance of missing strains of *D. nodosus* in the sample, and in the instance of this study, no facility was available in Victoria for the virotyping of isolates, so transport was required. This increases the likelihood of damage to the bacteria in transport and may affect the expression of proteases, the amount made or the ability to reliably have thermostability measured [17]. This may have been the cause of the disagreements from one sample in the clinically healthy data, and two from the clinically affected data. Despite this, there was no significant difference between the two tests when it came to identifying the virulence of the isolates. Instances of results where the rtPCR has detected both virulent and benign protease from a sample, yet culturing missed one or the other strain, are shown in 6 cases from the clinically affected data.

There are many challenges with footrot and the assessment of new diagnostic testing methods due to complex interplay between *D. nodosus*, environment and the host which may result in clinical signs of disease [18]. The method of assessment here reflects the way implementation and sample collection would occur in the field, and so the analysis is appropriate for Victoria’s methods of disease investigation. The most prominent difficulty is that the development of lesions is required for a visual diagnosis, to which rtPCR detection rate has been evaluated against. Factors like sheep breed, management, weather, and timing of inspection may all contribute to the lack of lesion, yet *D. nodosus* may still be present and found by the rtPCR. This would contribute to reduced specificity due to being rtPCR positive for *D. nodosus*, yet lesion negative, or lesions that are healing and not indicative of the infecting strain [19, 20].

## Conclusions

The improved speed and detection of virulent *D. nodosus* by this rtPCR assay could lead to a change of animal husbandry practices if the focus were to shift from clinical disease to the detection of virulent *D. nodosus*, indicating infection [15]. The ability to pool samples for this type of rtPCR has also been demonstrated [21, 22], an advantage in time and cost over culturing. Testing of this nature is also capable of detecting and quantifying the bacteria associated with the clinical disease, providing the basis to measure the success of various management practices for both treatment and prevention of footrot [23].

## Additional file

Additional file 1: Definition of footrot scoring system. (DOCX 12 kb)

## Abbreviations

EDTA: Ethylenediaminetetraacetic acid
rtPCR: Competitive real-time PCR
