# Supplementary material for: Assessment of a rtPCR for the detection of virulent and benign Dichelobacter nodosus, the causative agent of ovine footrot, in Australia
Source: BMC Vet Res. 2018 Aug 29;14:252. doi: 10.1186/s12917-018-1575-0 (PMC6114850; doi:10.1186/s12917-018-1575-0)
Supplement: Supplementary file 1 — Definition of footrot scoring system. (DOCX 12 kb) [file 12917_2018_1575_MOESM1_ESM.docx]

**Additional File 1. Definition of footrot scoring system.**

| **Score** | **Description** | **Additional description** |
| --- | --- | --- |
| 1 | Slight to moderate inflammation confined to interdigital  skin and involving erosion of the epithelium. | |
| 2 | Necrotising inflammation of interdigital skin which  also involves part or all of the soft horn of the axial  wall of the digit. | |
| 3 | Necrotising inflammation  with underrunning of part  or all of the soft horn of the  heel or sole which does not  extend to the abaxial edge  of the sole of the hoof. | 3a: Separation at skin-horn  junction with underrunning  extending no more than 5 mm across sole. |
|  |  | 3b: Underrunning no more than halfway across heel or sole. |
|  |  | 3c: Extensive underrunning of heel or sole but not extending to abaxial edge of the sole. |
| 4 | Underrunning extending to abaxial edge of sole. | |
| 5 | Necrosis of deeper epidermal layers (laminae) of  abaxial wall with under-running of hard horn. | |
